# Efficient and Rapid Induction of Human iPSCs/ESCs into Nephrogenic Intermediate Mesoderm Using Small Molecule-Based Differentiation Methods

**DOI:** 10.1371/journal.pone.0084881

**Published:** 2014-01-15

**Authors:** Toshikazu Araoka, Shin-ichi Mae, Yuko Kurose, Motonari Uesugi, Akira Ohta, Shinya Yamanaka, Kenji Osafune

**Affiliations:** 1 Center for iPS Cell Research and Application (CiRA), Kyoto University, Kyoto, Japan; 2 Institute for Integrated Cell–Material Sciences, Kyoto University, Kyoto, Japan; 3 Institute for Chemical Research, Kyoto University, Kyoto, Japan; 4 Gladstone Institute of Cardiovascular Disease, San Francisco, California, United States of America; Kanazawa University, Japan

## Abstract

The first step in developing regenerative medicine approaches to treat renal diseases using pluripotent stem cells must be the generation of intermediate mesoderm (IM), an embryonic germ layer that gives rise to kidneys. In order to achieve this goal, establishing an efficient, stable and low-cost method for differentiating IM cells using small molecules is required. In this study, we identified two retinoids, AM580 and TTNPB, as potent IM inducers by high-throughput chemical screening, and established rapid (five days) and efficient (80% induction rate) IM differentiation from human iPSCs using only two small molecules: a Wnt pathway activator, CHIR99021, combined with either AM580 or TTNPB. The resulting human IM cells showed the ability to differentiate into multiple cell types that constitute adult kidneys, and to form renal tubule-like structures. These small molecule differentiation methods can bypass the mesendoderm step, directly inducing IM cells by activating Wnt, retinoic acid (RA), and bone morphogenetic protein (BMP) pathways. Such methods are powerful tools for studying kidney development and may potentially provide cell sources to generate renal lineage cells for regenerative therapy.

## Introduction

Chronic kidney disease (CKD) is increasingly recognized as a global public health problem. Increased prevalence of CKD has led to a rise in the number of dialysis patients, and is associated with elevated morbidity and mortality due to the increased risk of cardiovascular diseases [1]–[3]. Most patients with CKD never recover their renal function, and there is a worldwide shortage of donor kidneys for transplantation; therefore, it is important to develop kidney regeneration therapy using embryonic stem cells (ESCs) [4]–[6] or induced pluripotent stem cells (iPSCs) [7]–[9], which have unlimited self-renewal capabilities and the potential to differentiate into any cell type in the body. However, directed differentiation methods from human ESCs (hESCs) or iPSCs (hiPSCs) into kidney lineage cells have not been fully developed.

Kidneys are derived from an early embryonic germ layer, the intermediate mesoderm (IM). In vertebrates, the IM sequentially develops into three stages of kidneys; the pronephros, mesonephros and metanephros. The mammalian adult kidney (metanephros) is formed by a reciprocal interaction between two precursor tissues, the metanephric mesenchyme and the ureteric bud [10]–[13]. Kidney regeneration methods that mimic normal development would first differentiate ESCs or iPSCs into IM, followed by formation of renal progenitors, such as the metanephric mesenchyme and ureteric bud, and eventually produce the various types of fully differentiated renal cells.

Previous research on kidney development in a mouse model showed that expression of a transcriptional regulator, *Odd-skipped related 1* (*Osr1*), starts early in the embryonic day (E) 7.5 IM from which the renal structures are derived [14], and is maintained until kidney organogenesis occurs. *Osr1* knockout mice lack renal structures, due to the failure to form the IM [15], [16]. Therefore, differentiation of pluripotent stem cells (PSCs) into *Osr1*-expressing IM cells is the first and crucial step toward the induction of renal lineage cells.

We recently established a highly efficient method for inducing the differentiation of IM cells from human iPSCs/ESCs (hiPSCs/ESCs), in which the combination of activin A and CHIR99021, a glycogen synthase kinase 3β inhibitor, is first used to generate mesendoderm [17], [18], followed by combined treatment with bone morphogenetic protein (BMP)-7 and CHIR99021 [19]. This protocol induces development of hiPSCs into OSR1^+^ IM cells at rate higher than 90%. The OSR1^+^ IM cells can further differentiate into multiple cell types constituting IM-derived organs, such as the kidneys, adrenal cortex, and genitalia, both *in vitro* and *in vivo* [19]. Although this method efficiently generates IM cells, modifications are needed for clinical application in kidney regeneration therapy. Growth factors, such as activin A and BMP-7, are expensive, and their effects are inconsistent among different lots. Differentiation methods using low-molecular-weight compounds would be less expensive and more consistent. Small molecules can control cellular processes by modulating signal transduction pathways, gene expression, or metabolism. High-throughput screening has been previously used to identify novel small molecules that support the directed differentiation of mouse ESCs (mESCs) and hESCs into definitive endoderm [20], pancreatic progenitors [21], and cardiomyocytes [22].

*In vitro* differentiation of the undifferentiated cell mass in the fertilized eggs of amphibians such as Xenopus *laevis* [23]–[25], mESCs [26]–[29] and hESCs/iPSCs [30], [31] into renal lineages has been stimulated using retinoic acid (RA). However, the roles and mechanisms of RA signaling have remained unclear. Previous studies reported that the signaling pathways of BMP-2 or BMP-4 were involved in the regulation of mesoderm formation, including differentiation of the IM in chick embryos [32], [33], but little is known about the developmental mechanisms of IM formation, especially in mammals.

In the present study, we aimed to develop an efficient, stable and low-cost method for differentiating hiPSCs/ESCs into IM cells using small molecules. We performed high-throughput screening of approximately 1,800 chemical compounds and identified two retinoic acid receptor (RAR) agonists, AM580 and TTNPB, that efficiently induce the differentiation of hiPSCs/ESCs into IM cells. Based on these results, we established a simple, rapid, and highly efficient differentiation method, using a combination of just two chemicals (CHIR99021 with TTNPB or AM580), to induce development of hiPSCs/ESCs into IM cells that have the potential to give rise to IM derivatives. We found that combined treatment with CHIR99021 and TTNPB or AM580 induces the differentiation of IM cells by activating the expression of BMP-4 through RAR. We further demonstrated that IM cells can be directly generated from hiPSCs/ESCs without the mesendoderm step, by activating the Wnt, RA, and BMP signaling pathways. The new differentiation method using small molecules provides less expensive and more consistent means of generating IM cells than the growth factor method. Therefore, this new method is useful for studying the mechanisms of mesoderm and kidney development and may provide a source of renal lineage cells for use in regenerative medicine to treat CKD.

## Materials and Methods

### Cell Culture

Five hiPSC lines (201B6, 253G1, 253G4, 585A1 and an OSR1-green fluorescent protein (GFP) knock-in hiPSC line, 3D45, generated from 201B7 [7], [19], [34], [35]), and three hESC lines (H9, khES1 and khES3 [6], [36]), were routinely cultured on feeder layers of mitomycin C-treated mouse SNL feeder cells [37] in Primate ES medium (ReproCELL) supplemented with 500 U/ml penicillin/streptomycin (Invitrogen) and 4 ng/ml recombinant human basic fibroblast growth factor (bFGF, Wako). Cells were split at a ratio of 1∶3–1∶6 every six to eight days using CTK dissociation solution consisting of 0.25% trypsin (Invitrogen), 0.1% collagenase IV (Invitrogen), 20% Knockout serum replacement (KSR, Invitrogen) and 1 mM CaCl_2_ in PBS [7].

### Chemicals Libraries

The library included compounds from the Prestwick Chemical library (Prestwick Chemical, Illkirch, France) and the ENZO library (ENZO Life Sciences, Farmingdale, NY, USA).

### High-throughput Chemical Screening

An OSR1-GFP knock-in hiPSC line, 3D45 [19], grown on mouse embryonic fibroblast (MEF) feeder cells from E 12.5 ICR mouse embryos, were dissociated by an enzymatic method with CTK dissociation solution. After washing them with PBS (Nakalai Tesque), the cells were scraped off with a cell scraper, dissociated by pipetting and seeded on Human Collagen Type I-coated 96-well plates (BD) with MEF-conditioned Primate ES medium containing 10 ng/ml bFGF. When hiPSC colonies attained approximately 70% confluency, the cells were treated with 100 ng/ml recombinant human/mouse/rat activin A (R&D Systems), 3M CHIR99021 (Wako) and 10 µM Y27632 (Wako) in Stage 1 medium containing DMEM/F12+Glutamax (Invitrogen) supplemented with 500 U/ml penicillin/streptomycin, 2% FBS (HyClone) and 10 µM Y27632 for two days. Then, the compound treatments were performed at a final concentration of 1 µM in 100 µl of Stage 2 medium containing DMEM/F12+Glutamax supplemented with 0.1 mM non-essential amino acids (Invitrogen), 500 U/ml penicillin/streptomycin, 0.55 mM 2-mercaptoethanol (Invitrogen) and 10% KSR supplemented with 0.1% DMSO (v/v) per well. After five days of compound treatments, the cells were dissociated by trypsinization, resuspended in HBSS with propidium iodide (1∶1000, Wako) and examined for their GFP expression by a LSR Fortessa equipped with a High Throughput Sampler (BD). Positive hits were defined as the compounds that induced GFP^+^ cells at three standard deviations (3SD) above the DMSO controls without producing autofluorescence or cytotoxicity.

### Generation of Efficacy Curves

The OSR1-GFP knock-in hiPSCs (3D45) were dissociated and plated onto Matrigel-coated 96-well plates in Stage 1 medium with 100 ng/ml activin A, 3 µM CHIR99021 and 10 µM Y27632. After two days of Stage 1 treatment, AM580 or TTNPB was added at a final concentration of 10 µM, 5 µM, 2.5 µM, 1.2 µM, 600 nM, 300 nM, 150 nM, 75 nM, 37.5 nM, 19 nM, 10 nM, 5 nM, 2.5 nM, 1.2 nM, 0.6 nM or 0.3 nM. Five days later, the induction of OSR1^+^ cells was detected by flow cytometry. For the replacement of TTNPB or AM580 in the small molecule method, all-trans retinoic acid (ATRA), adapalene, or CD1530 was added at a final concentration of 10 µM, 5 µM, 2.5 µM, 1.2 µM, 600 nM, 300 nM, 150 nM, 75 nM, 37.5 nM, 19 nM, 10 nM, 5 nM or 2.5 nM, and BMS493, LE135, MM11253 or SR11237 was used at a final concentration of 10 µM, 5 µM, 2.5 µM, 1.0 µM, 100 nM, 10 nM, 1.0 nM or 0.1 nM. The induction of OSR1^+^ cells was analyzed by flow cytometry on day 6 of treatment. For the addition of SR11237 or UVI3003 to the TTNPB method, these chemicals were added at final concentrations of 5 µM, 2.5 µM, 1.0 µM, 100 nM, 10 nM or 1.0 nM. The details of the growth factors and chemical compounds used in this study are shown in Table S4.

### Differentiation Methods

To induce IM cells, hiPSC/ESC colonies grown on feeder layers of mitomycin C-treated mouse SNL feeder cells [37] were first dissociated by an enzymatic method with CTK dissociation solution, and incubated on gelatin-coated plates for 30 min to remove SNLs. Then, the cells were dissociated into single cells by gentle pipetting after the treatment with Accutase (Innovative Cell Technologies, Inc.) for 20 min. The cells were then seeded on Matrigel (Matrigel Matrix Growth Factor Reduced, BD)-coated plates at a density of 1.5×10^5^ cells/cm^2^.

For the original small molecule methods, the dissociated hiPSCs/ESCs were treated with 3 µM CHIR99021 and 1 µM AM580 (Santa Cruz Biotechnology) or 1 µM TTNPB (Santa Cruz Biotechnology) in Stage 1 medium for two days. Next, the culture medium was replaced with Stage 2 medium containing 1 µM AM580 or 1 µM TTNPB. The Stage 2 cultures were maintained for an additional three to 12 days. The growth factor method was previously described as a “single-cell method” [19].

For the serum-free small molecule methods, the cells were cultured with a serum-free medium containing DMEM/F12 + Glutamax supplemented with 1×B27 supplement (Invitrogen) and 500 U/ml penicillin/streptomycin on Synthemax (Synthemax II-SC Substrate, Corning)-coated plates throughout the differentiation culture.

### Flow Cytometry and Cell Sorting

The cells were treated with 0.25% trypsin/EDTA for 5 min at 37°C, and dissociated by pipetting in HBSS (GIBCO). Dead cells stained with propidium iodide were excluded from the analysis. The cells were analyzed and sorted using a FACS Aria II cell sorter (BD). The isolated cells were collected in PBS with 2% FBS containing 10 µM Y27632.

### RT-PCR and Real-time Quantitative RT-PCR (qRT-PCR)

Total RNA was isolated from triplicate samples in three independent experiments using an RNeasy kit (Qiagen) according to the manufacturer's recommendations, followed by cDNA synthesis using standard protocols. Briefly, first-strand cDNA was synthesized from 1 µg of total RNA using ReverTra Ace (TOYOBO). The cDNA samples were subjected to PCR amplification using a thermal cycler (Veriti 96 well Thermal Cycler, Applied Biosystems). PCR was performed using the Ex-Taq PCR kit (Takara) according to the manufacturer's instructions. The PCR cycles were as follows: for *β-ACTIN*, initial denaturation was performed at 94°C for 2.5 min, followed by 25 cycles of 94°C for 30 s, 60°C for 1 min, 72°C for 30 s, and a final extension at 72°C for 10 min. For the other genes, the cycles consisted of initial denaturation at 94°C for 2.5 min, followed by 30–40 cycles of 94°C for 30 s, 58–62°C for 30 s, 72°C for 30 s, and final extension at 72°C for 7 min. mRNA from human fetal kidney was used as a positive control for expression of *OSR1*, *PAX2*, *LIM1*, *WT1*, *EYA1*, *SALL1*, and *CITED2*. mRNA from human adult ovaries was used as a positive control for *SOX17*. mRNA from neural progenitor cells, induced from hiPSCs by SFEBq culture [38], was used as a positive control for *SOX1*. mRNA from human umbilical vein endothelial cells (HUVECs) was used as a positive control for *KDR*. mRNA from human adult cervix was used as a positive control for *SIX2*. mRNA from human adult kidney was used as a positive control for *HOXD11*, *FOXD1*, *SALL4*, and *HOXB7*. mRNA from human adult ovaries was used as a positive control for *GATA4* and *GATA6*. mRNA from human adult adrenal gland was used as a positive control for *SF1* and *HSD3β2*. mRNA from human adult testes was used as a positive control for *DAX1* and *LHX9*. qPCR was performed using the Step One Plus Real-Time PCR System (Applied Biosystems) and the SYBR Green PCR Master Mix (Takara). Denaturation was performed at 95°C for 30 s, followed by 45 cycles at 95°C for 5 s and at 60°C for 30 s. The threshold cycle method was used to analyze the data for the gene expression levels as recommended by the manufacturer, and was calibrated to the levels of the housekeeping gene, *β-ACTIN*. The PCR reactions were performed in triplicate for each sample. The primer sets are shown in Table S2.

### Immunostaining

The cells were fixed with 4% paraformaldehyde (PFA, Nacalai Tesque)/PBS for 20 min at 4°C. For immunostaining of the cytoplasm, the cells were washed with PBS and blocked with 5% normal donkey serum (Chemicon)/PBST (PBS/0.1% Triton X-100, Nacalai Tesque) for 1 hr at room temperature. The primary antibodies were diluted in blocking solutions and incubated with samples overnight at 4°C. For nuclear immunostaining, the cells were washed with PBS and incubated in PBS/0.2% Tween 20 (Wako) for 30 min at room temperature, followed by blocking with 3% normal donkey serum/1% BSA (Nacalai Tesque)/PBST (PBS/0.25% Triton X-100) for 1 hr at room temperature. The primary antibodies were diluted in blocking solution and incubated with samples overnight at 4°C. Secondary antibodies were diluted in blocking solution and incubated with samples for 1 hr at room temperature. The details of the antibodies used in this study are shown in Table S3.

### *In Vitro* Differentiation Culture of OSR1^+^ Cells

The OSR1^+^ IM cells induced with the TTNPB method were isolated by flow cytometry sorting on culture day 6, seeded onto gelatin-coated 96-well plates at a density of 1.0×10^5^ cells/well, and cultured with Stage 2 medium containing 10 µM Y27632, 100 ng/ml recombinant human BMP-7 and 100 ng/ml recombinant mouse Wnt3a or 1 µM CHIR99021 [19]. After an additional eight days of culture, the cells were examined by RT-PCR and immunostaining.

### Graft Preparation and Implantation

The hiPSC-derived OSR1^+^ on culture day 6 was isolated by flow cytometry sorting, seeded onto low attachment 96-well plates (Lipidure Coat, NOF Corp) at a density of 1.0×10^5^ cells per well, and cultured with Stage 2 medium containing 10 µM Y27632 for 2 days. Then, about 20 aggregates were transferred onto polyethylene terephthalate fiber-Collagen Sponge (MedGEL) that was prewetted with Stage 2 medium. The aggregates and sponge were overlayed with 50 l of Matrigel. The resultant implant constructs were placed in an incubator set at 37°C and 5% CO_2_ for 1 h to allow the construct to gel. These constructs were transferred to culture dishes with prewarmed medium until implantation. One of the epididymal fat pads (EFPs) of immunodeficient mice (NOD. CB17-*Prkd* ^scid^/J) was carefully externalized through an abdominal incision, then a single implant construct was wrapped in the EFP and the implanted EFP were returned to abdominal cavity. After 4 weeks of implantation, mice were killed and the serial sections of implanted tissues were examined with immunostaining [19].

### Aggregate Culture of OSR1^+^ Cells

The day 6 isolated OSR1^+^ IM cells differentiated with the TTNPB method were quickly seeded onto 96-well low-cell-adhesion plates (Lipidure Coat, NOF Corp) with Stage 2 medium (1.0×10^5^ cells in 100 l/well) containing 10 M Y27632, and then were cultured at 37°C to form cellular aggregates. After eight days of suspension culture, the aggregates were fixed, and serial sections were examined by immunostaining. Stained sections were analyzed with a LSN710 confocal microscope (Zeiss).

### Organ Culture Experiments

The organ culture experiments were performed as described previously [19], [39]. Briefly, E 11.5 metanephric kidneys from ICR mice were dissected in Improved MEM (Invitrogen). The metanephric kidneys were then placed in 0.05% trypsin-EDTA (GIBCO) for 10 min at 37°C and dissociated by pipetting. The dissociated cells were stabilized in Improved MEM supplemented with 500 U/ml penicillin/streptomycin and 10% FBS (Kidney culture medium, KCM) for 10 min at 37°C, and then were filtered through a 40 m cell strainer (BD). A total of 1.0×10^5^ freshly dissociated metanephric cells and 1.0×10^4^ OSR1^+^ cells isolated by flow cytometry sorting after the induction with the small molecule methods were mixed, seeded onto 96-well low-cell-adhesion plates with 10 M Y27632, then cultured overnight at 37°C to form aggregates. The cellular aggregates were then cultured at the air-fluid interface on 0.4 m pore polycarbonate filters (Millipore) supplied with KCM at 37°C. After eight days of organ culture, the aggregates were fixed, and serial sections were examined by immunostaining.

### Statistical analysis

The statistical significance of the differences in the frequency of renal tubule formation between the hiPSC-derived OSR1^+^ cells generated with small molecule treatments and the control undifferentiated hiPSCs in organ culture was analyzed using Fisher's exact test.

### Animal Welfare

This study was carried out in strict accordance with the recommendations in the Regulation on Animal Experimentation at Kyoto University. The protocol in this study was approved by the Animal Research Committee of Kyoto University. All efforts were made to minimize suffering. Mice were humanely sacrificed prior to tissue collection.

### Inhibitor Screening

The effects of 18 different chemical inhibitors on the induction of OSR1^+^ IM cells with the TTNPB method were examined at different concentrations, as indicated in Figure S4. After 72 hrs of treatment, the cells were examined by the same procedures used for the high-throughput chemical screening described above. The details of the growth factors and chemical compounds used in this study are shown in Table S4.

### Western Blot Analysis

The cells treated with the small molecule methods were harvested in lysis buffer (BioVision). Proteins were separated by 10% Tris-Glycine SDS/PAGE (BIO-RAD) under denaturing conditions and transferred to a PVDF membrane (BIO-RAD). After blocking with 5% skim milk in PBS/0.1% Tween20, the membrane was incubated with antibodies against phospho-Smad1/5 or β-actin overnight at 4°C. The membrane was then washed, incubated with anti-mouse/rabbit peroxidase-conjugated secondary antibody (Jackson ImmunoResearch) at room temperature for 1 hr and developed by a chemiluminescence detection system (GE Healthcare).

### Gene Knockdown Experiments

The dissociated OSR1-GFP knock-in hiPSCs (3.0×10^4^ cells in 0.5 ml/well of 24-well plates) in Stage 1 medium with 3 M CHIR99021, 1 M TTNPB, and 10 M Y27632 were transfected with 50 nM siRNA for *RARB* (MISSION siRNA, Sigma) or Universal Negative Control siRNA Duplex (Stealth RNAi Negative Control Kit, Invitrogen) using the Lipofectamine RNAiMAX reagent (Invitrogen) throughout Stage 1. After 48 hrs of Stage 1 treatment, the cells were transfected again, this time with Stage 2 medium containing 1 M TTNPB and the same siRNA solutions used in Stage 1.

## Results

### High-throughput Screening Identified Small Molecules that Induce Production of IM Cells from hiPSCs

**Figure 1:**
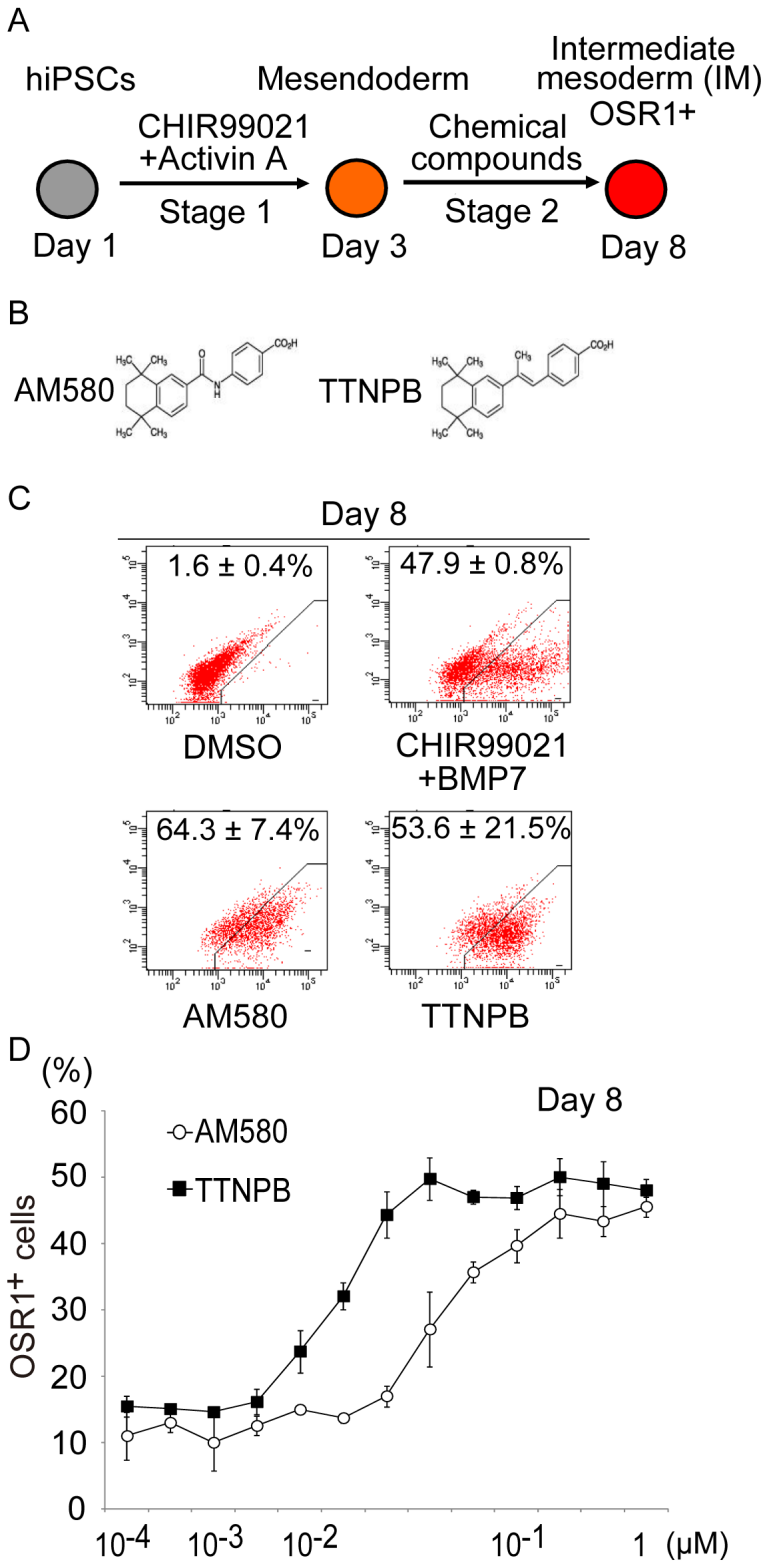
High-throughput Screening and Characterization of the Two Hit Compounds. (A) Schematic diagram of the screening strategy to induce the differentiation of human iPSCs (hiPSCs) into intermediate mesoderm (IM). Stage 1 indicates the differentiation step from hiPSCs into mesendoderm. Stage 2 indicates differentiation from mesendoderm into IM. Chemical screening was performed on Stage 2 cells. (B) The chemical structures of the two hit compounds, AM580 and TTNPB. (C) Results of flow cytometric analyses of the induction of OSR1^+^ cells on culture day 8; treatments consisted of two days of Stage 1 with CHIR99021 and activin A, and five days of Stage 2 with AM580 or TTNPB, DMSO (negative control), or combined CHIR99021 and BMP7 (positive control). (D) Dose-response curve of OSR1^+^ cell induction on day 8 by AM580 and TTNPB. The data in (C) and (D) are means±SD of three independent experiments (n = 3).

The screen used an hiPSC reporter line, in which the GFP coding sequence had been knocked-in to the OSR1 gene locus (OSR1-GFP knock-in hiPSC line, 3D45; [19]) and flow cytometry to quantitatively identify small molecules that increase the induction rate of OSR1^+^ cells. Mesendoderm cells, the starting material for the screen, were generated from hiPSCs (3D45 cells) using a combination of 100 ng/ml activin A and 3 µM CHIR99021 for two days (Figure 1A, Stage 1), as described previously [19]. The mesendoderm cells were then treated with individual compounds for an additional five days (Stage 2). We screened two chemical libraries, the Prestwick Chemical library and ENZO library, comprising a total of 1,821 bioactive compounds and known drugs. Positive hits were defined as compounds that induced OSR1 (GFP)^+^ cells at three standard deviations (3SD) above the negative control (DMSO), without producing autofluorescence or cytotoxicity. The OSR1^+^ IM cells induced by the combination of CHIR99021 and BMP-7, which we described previously [19], were used as a positive control.

The screening identified two compounds, AM580 and TTNPB, both known to be RAR agonists, as inducers of OSR1^+^ cells (Figure 1B). The induction rates of OSR1^+^ cells in populations treated with AM580 (64.3±7.4%, n = 3) or TTNPB (53.6±21.5%, n = 3) were higher than rates for DMSO-treated controls (1.6±0.4%, n = 3) and positive controls (47.9±0.8%, n = 3) (Figure 1C).

Additional experiments were carried out to optimize the concentrations of the compounds. Titration curves from 0.3 nM to 1.2 M for AM580 and TTNPB showed that both compounds induced OSR1^+^ cells in a dose-dependent manner, with the highest efficiencies at 300 nM to 1.2 M for AM580, and at 37.5 nM to 1.2 M for TTNPB (Figure 1D).

### A Small Molecule-based Differentiation Method Rapidly and Efficiently Produces IM Cells from hiPSCs

**Figure 2:**
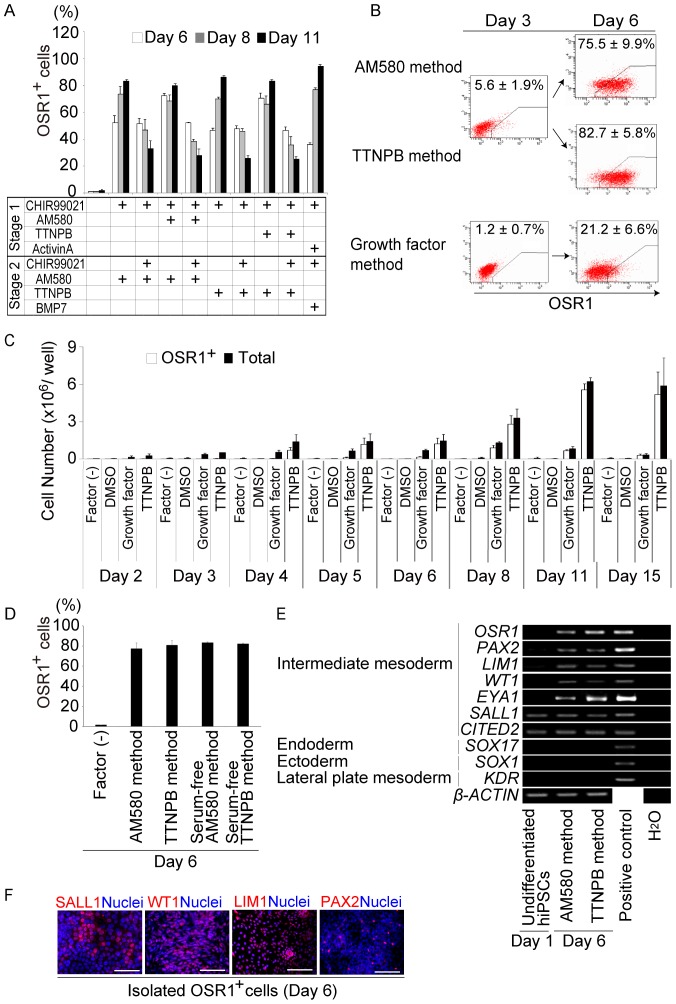
The Small Molecule Method for Differentiating hiPSCs into IM Cells. (A) The results of the flow cytometric analyses comparing the induction efficiency of OSR1^+^ cells on culture days 6, 8, and 11 for various treatments with CHIR99021 and AM580 or TTNPB, and the growth factor method (CHIR99021 and activin A during Stage 1, CHIR99021 and BMP7 during Stage 2). (B) Results of flow cytometric analyses of OSR1^+^ cell induction on days 3 and 6 of the AM580, TTNPB, and growth factor methods. (C) Numbers of OSR1^+^ and total cells generated by the TTNPB and growth factor methods. (D) Results of the flow cytometric analyses showing the differentiation of OSR1^+^ cells on day 6 using the serum-free small molecules methods. (E) Results of RT-PCR analyses showing mRNA expression of IM and non-IM marker genes in undifferentiated hiPSCs before treatment on day 1, and in OSR1^+^ cells on day 6 after induction by the small molecule method. (F) Immunostaining for SALL1, WT1, LIM1, and PAX2 in the OSR1^+^ cells on day 6 of induction by the TTNPB method. Scale bars, 100 m. The data in (A–D) are means±SD of three independent experiments (n = 3). The data in (E) and (F) are representative of three independent experiments. See Figure S2 for additional data.

Previous studies reported that the activation of Wnt signaling by recombinant Wnt proteins or chemicals resulted in the differentiation of mESCs and hESCs into mesendoderm without activin A treatment [40], [41]. Results of the present study confirmed that mesendoderm cells were induced from hiPSCs by the treatment with CHIR99021 alone (Figures S1A and B). To further optimize the method for generating OSR1^+^ cells, we examined various combinations of CHIR99021 with AM580 or TTNPB, without activin A (Figure 2A). We found that, when hiPSCs were treated with 3 M CHIR99021 combined with 1 M AM580 or 1 M TTNPB for two days during Stage 1, and with 1 M AM580 or 1 M TTNPB for an additional three days during Stage 2, the induction rate of OSR1^+^ cells on day 6 was increased to around 80% (Small molecule method, including AM580 and TTNPB methods, Figure 2B and Figure S2A). The numbers of OSR1^+^ and total cells peaked on culture day 11 (Figure 2C and Figure S2B). The small molecule method induced a much larger number of OSR1^+^ cells than our growth factor method [19] at all of the tested time points. Therefore, we established a rapid (requiring only five days), highly efficient (approximately 80%) and robust differentiation method for generating OSR1^+^ cells from hiPSCs using only two chemicals.

The development of a well-defined method for differentiating PSCs would provide an excellent system that could be used to elucidate the molecular mechanisms of IM differentiation. We therefore devised a serum-free differentiation method by replacing both fetal bovine serum (FBS) used in Stage 1 and KSR in Stage 2 of the small molecule method with B27 supplement. Moreover, Matrigel which coated dishes was replaced by Synthemax, xeno-free coating material. The induction efficiency of OSR1^+^ cells in the refined protocol (Serum-free small molecule method, including Serum-free AM580 and TTNPB methods) was about 80% and comparable to the original small molecule method (Figure 2D).

To confirm that the human OSR1^+^ cells induced by the small molecule method have an IM signature, we used RT-PCR analyses and immunocytochemistry to examine the expression of multiple IM marker genes. After five days of treatment, the OSR1^+^ cells isolated by flow cytometry sorting expressed *PAX2*, *LIM1*, *WT1*, *EYA1*, *SALL1*, and *CITED2* (Figure 2E), which are all known to be expressed in cells of IM or kidney lineage [12], [14]. Moreover, some of the OSR1^+^ cells isolated on culture day 6 were positively stained for SALL1, WT1, LIM1, or PAX2 (Figure 2F). In contrast, markers for non-IM lineage cells, including *SOX17* for endoderm, *SOX1* for ectoderm, and *KDR* for lateral plate mesoderm, were not detected (Figure 2E). Most of the OSR1^+^ cells produced by the small molecule method appear to represent the IM.

As it has been demonstrated that different hiPSC/ESC lines vary in their differentiation potential [34], [42], we examined effectiveness of the small molecule method using multiple hiPSC/ESC lines (Figure S3) [6], [7], [34]–[36]. The results confirmed that the method works with dermal fibroblast-derived iPSC lines (201B6, 253G1, and 253G4), a peripheral blood cell-derived iPSC line (585A1), and hESC lines (KhES1, KhES3, and H9), in addition to the 3D45 cells (201B7).

### IM Cells Generated with the Small Molecule Method Can Differentiate into IM Derivatives and Form Renal Tubule-like Structures

**Figure 3:**
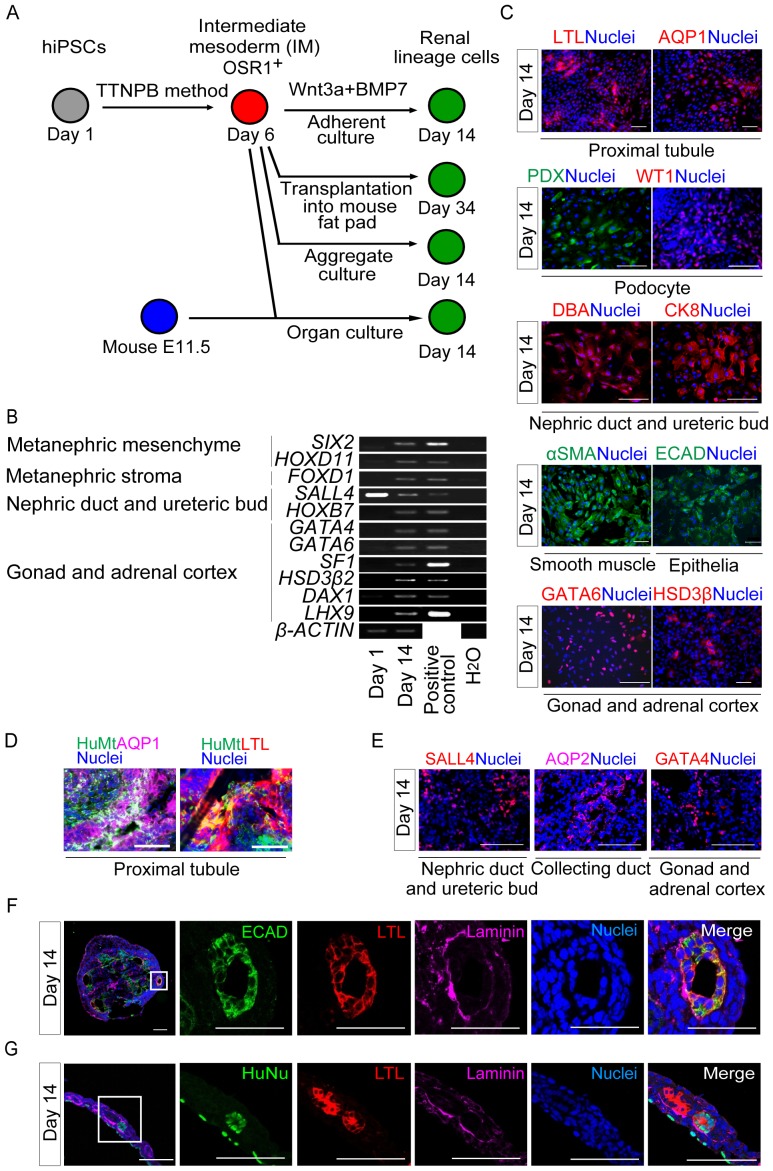
Developmental Potential of hiPSC-derived IM Cells Generated by the Small Molecule Method. (A) Schematic showing further *in vitro* and *in vivo* development of OSR1^+^ cells that were differentiated from OSR1-GFP knock-in hiPSCs (3D45), using the TTNPB method. (B) Results of RT-PCR analyses showing mRNA expression of marker genes for the developing kidney, gonad, and adrenal cortex in differentiated OSR1^+^ cells on culture day 14. (C) Differentiated cells stained on day 14 with antibodies or lectins against markers of IM derivatives: LTL and AQP1 for the proximal renal tubule, PDX and WT1 for glomerular podocytes, DBA and CK8 for the nephric duct and ureteric bud, &SMA for smooth muscle, ECAD as an epithelial marker, and GATA6 and HSD3β as markers of gonad or adrenal cortex. (D) Immunostaining of histological sections of four-week-old hiPSC-derived IM grafts generated by the TTNPB method transplanted into immunodeficient mice (NOD. CB17-*Prkdc* ^scid^/J) for human mitochondria (HuMt) (green), LTL (red), AQP1 (purple) and all nuclei (blue). (E) Immunostaining for IM derivative markers (SALL4 for the nephric duct and ureteric bud, AQP2 for the collecting duct, and GATA4 for the gonads or adrenal cortex) of histological sections of cell aggregates generated from OSR1^+^ cells isolated on day 6 and grown in suspension culture for an additional 8 days. (F) Renal tubule-like structures formed inside the cell aggregates on day 14 stained for ECAD (green), LTL (red), laminin (purple), and nuclei (blue). The five panels on the right are magnified views of the solid box in the left panel. (G) Section immunostaining of organ culture samples collected on day 14, for human nuclei (HuNu, green), LTL (red), laminin (purple), and all nuclei (blue). The five panels on the right are magnified views of the solid box in the left panel. The data in (B, C), (D) and (E, F) are representative of the findings of three, five and four independent experiments, respectively. Scale bars, 100 m.

Osr1^+^ IM cells contribute to a variety of cell types during embryonic development in mice, including cells of the adrenal cortex and gonad, as well as kidney [14]. We recently demonstrated that hiPSC-derived OSR1^+^ IM cells generated by the growth factor method differentiated into these cell types, following an additional seven-day treatment with 100 ng/ml BMP-7 and 100 ng/ml Wnt3a or 1–3 M CHIR99021 [19]. In the present study, we treated OSR1^+^ cells isolated on culture day 6 of the small molecule method with these factors for an additional eight days to examine their developmental potential to give rise to IM-derivative cell types (Figure 3A, upper panel). The treated cells expressed marker genes for IM-derivative cell types, including *SIX2* and *HOXD11* for the metanephric mesenchyme; *FOXD1* for metanephric stroma; *SALL4* and *HOXB7* for the nephric duct and ureteric bud; and *GATA4, GATA6*, *SF1*, *HSD3β2*, *DAX1* and *LHX9* for gonadal or adrenocortical cells (Figure 3B). The differentiated cells also stained positively for a number of renal lineage markers: *Lotus tetragonolobus* lectin (LTL) and aquaporin 1 (AQP1) for the proximal renal tubule, podocalyxin (PDX) and WT1 for glomerular podocytes, *Dolichos biflorus* agglutinin (DBA) and cytokeratin 8 (CK8) for nephric duct and ureteric bud, αSMA for smooth muscle, E-cadherin (ECAD) for epithelia, and GATA6 and HSD3β for the gonad or adrenal cortex (Figure 3C).

Next, to assess the developmental potential of hiPSC-derived IM cells *in vivo*, we transplanted day 6 OSR1^+^ cells generated with the TTNPB method into the epididymal fat pads (EFPs) of immunodeficient mice (NOD. CB17-*Prkdc* ^scid^/J) (Figure 3A, upper middle panel). At four weeks after transplantation, grafts from five independent experiments contained cells immunoreactive for adult renal cell markers, such as AQP1 and LTL (Figure 3D), with no evidence of either teratomas or the formation of three-dimensional renal structures, which was similar to the findings obtained from the transplantation of hiPSC-derived IM cells generated with the growth factor method, as described previously [19].

To further evaluate the multi-lineage differentiation potential, cellular aggregates were generated from the OSR1^+^ IM cells, by culturing them in non-attachment dishes for an additional eight days with a ROCK inhibitor, Y27632, that increases the survival rate of dissociated hiPSCs/ESCs and the cells differentiated from them [34], [43] (Figure 3A, lower middle panel). The aggregates contained cells that were positively immunostained for IM-derivative markers, including SALL4 for the nephric duct and ureteric bud, AQP2 for the collecting duct, and GATA4 for the gonad and adrenal cortex (Figure 3E). Furthermore, renal tubule-like structures were formed inside the aggregates, which were positive for ECAD, LTL, and a polarized epithelial marker, laminin (Figure 3F).

The ability of the hiPSC-derived OSR1^+^ cells produced by the small molecule method to differentiate into three-dimensional renal structures was further examined by co-culturing them with mouse metanephric cells in an organ culture setting, as previously described using hiPSC-derived IM cells generated by the growth factor method [19], [39] (Figure 3A, lower panel). The human OSR1^+^ cells were integrated into the mouse metanephric tissues and differentiated into polarized tubule-like structures, which stained positively for both LTL and laminin (Figure 3G). We examined 145 organ culture samples: 37 with hiPSC-derived OSR1^+^ cells induced with AM580, 49 induced with TTNPB, and 59 controls with undifferentiated hiPSCs. Human LTL^+^/laminin^+^ renal tubule-like structures originated from the OSR1^+^ IM cells in 18 out of the 37 samples (48.6%) induced using AM580, and in 24 out of the 49 samples (49.0%) generated using TTNPB. On the other hand, undifferentiated hiPSCs co-cultured with mouse metanephric cells did not form any kidney-like structures or express any of the markers (0%, n = 59; AM580 vs. undifferentiated hiPSCs, *p*<0.001; TTNPB vs. undifferentiated hiPSCs, *p*<0.001). Thus, hiPSC-derived OSR1^+^ IM cells induced by the small molecule method appear to have the potential to differentiate into IM derivatives and to form three-dimensional renal structures.

These results suggest that the human IM cells generated with the small molecule method have the potential to become renal lineage cells both *in vitro* and *in vivo*, similar to the IM cells generated with the growth factor method [19].

### IM Differentiation by the Small Molecule Method Involves RARβ Signaling

**Figure 4:**
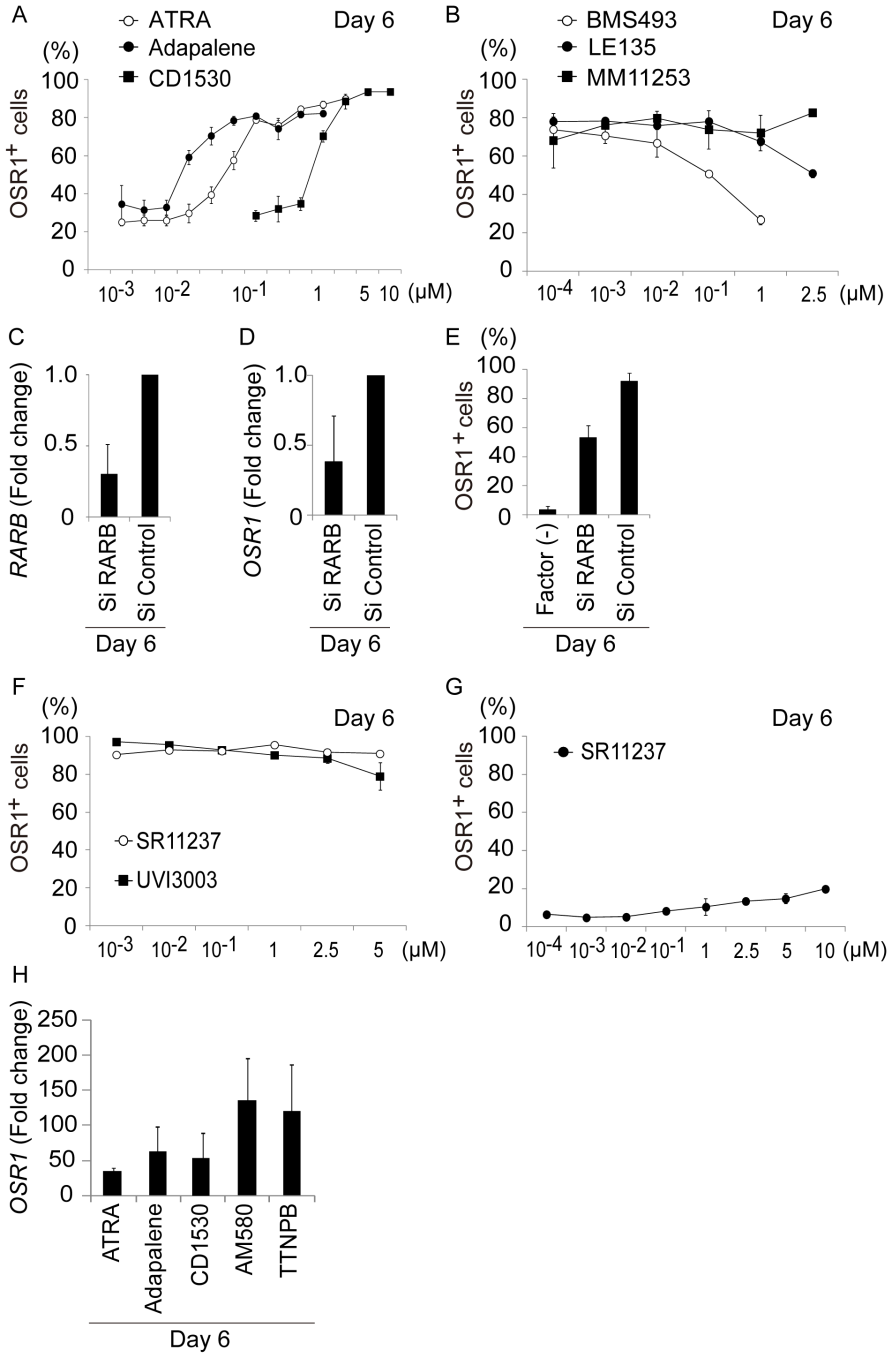
The Small Molecule Method Functions via RARβ Receptors. (A) Results of the flow cytometric analyses showing induction of OSR1^+^ cells by the small molecule method, when AM580 or TTNPB was replaced by all-trans retinoic acid (ATRA), adapalene, and CD1530. (B) Effects of adding RAR antagonists, BMS493, LE135, and MM11253, on the induction of OSR1^+^ cells by the TTNPB method. (C) The knockdown efficiency of siRNAs targeting *RARB*, and (D and E) effects on expression levels of *OSR1* and the induction of OSR1^+^ cells by the TTNPB method. (F) Effects of adding a pan-RXR agonist (SR11237) or a pan-RXR antagonist (UVI3003) to the induction efficiency of OSR1^+^ cells by the TTNPB method. (G) The induction of OSR1^+^ cells by the small molecule method, when SR11237 replaced AM580 or TTNPB. (H) Results of qRT-PCR analyses showing *OSR1* expression activated by the AM580 and TTNPB methods, and when ATRA, adapalene, or CD1530 were used instead of AM580 or TTNPB. OSR1-GFP knock-in hiPSCs prior to treatments were used to normalize the data. The data in (A–H) are presented as the mean±SD on culture day 6 of three independent experiments (n = 3).

Two families of RA nuclear receptors act as transcriptional transducers of retinoid signals during normal development: RA receptors (RARα, β, and γ) and retinoid X receptors (RXRα, β, and γ) [44], [45]. To determine which RAR and RXR isotypes are activated during human IM cell differentiation by the small molecule method, we examined the effects of three RAR agonists, ATRA, adapalene, and CD1530; three RAR antagonists, BMS493, LE135, and MM11253; one RXR agonist, SR11237; and one RXR antagonist, UVI3003, whose affinities to the RAR and RXR isotypes are concentration-dependent (Table S1) [46]–[49]. It has been reported that signals through RAR, but not through RXR, are involved in the mESC differentiation into IM cells [29]. In addition, TTNPB and AM580 are known to be RAR agonists, so we first examined the roles of RAR signaling pathways. Induction of OSR1^+^ cells when TTNPB or AM580 was replaced with adapalene (a RARβγ agonist) required concentrations greater than 10–75 nM, suggesting that the adapalene works mainly through RARβ or RARγ. Replacement of TTNPB or AM580 with CD1530, a selective RARγ agonist, required about 100 times higher concentration than adapalene to induce OSR1^+^ cells, indicating that signals through RARγ might not play central roles in IM differentiation (Figure 4A and Table S1). Together, these results suggest that the IM cell differentiation from hiPSCs in the small molecule method is primarily mediated by RARβ signaling.

To further analyze the roles of RAR isotypes, we added RAR antagonists to the TTNPB method: a pan-RAR antagonist, BMS493; a selective RARβ antagonist, LE135; and a selective RARγ antagonist, MM11253 (Figure 4B). Induction of OSR1^+^ cells was reduced by adding BMS493 or LE135, but not by adding MM111235, confirming the involvement of RARβ signaling in IM cell differentiation.

We also examined the effects of small-interfering RNA (siRNA) targeting RARβ on the induction of IM cells in the TTNPB method. The addition of siRNA for RARβ, which silenced around 70% of *RARβ* expression, reduced *OSR1* gene expression by approximately 70%, compared to cells treated with control siRNA (Figures 4C and D). Consistent with these findings, flow cytometry analyses showed that induction of OSR1^+^ cells decreased by approximately 40% after transfection of the siRNA for RARβ (Figure 4E). Thus, signals through RARβ are essential for human IM cell differentiation by the small molecule method.

To elucidate the roles of RXR signals in the development of human IM cells, we examined effects of a pan-RXR agonist, SR11237, and a pan-RXR antagonist, UVI3003, on the TTNPB method. Addition of either compound did not significantly change the induction of OSR1^+^ cells (Figure 4F). However, when SR11237 was used instead of TTNPB or AM580, the induction rate of OSR1^+^ cells was less than 20% (Figure 4G). Therefore, IM differentiation from hiPSCs does not appear to be regulated by signaling through RXR receptors.

To determine which RAR agonists are most effective for inducing IM cell differentiation, we compared the efficacy of the three RAR agonists (ATRA, adapalene, and CD1530) with AM580 and TTNPB. As induction of OSR1^+^ cells peaked at 150 nM to 2.5 M ATRA, 150 nM to 1.2 M adapalene, and 5–10 M CD1530, when those compounds replaced TTNPB or AM580 (Figure 4A), we used 1 M ATRA, adapalene, AM580, and TTNPB, and 5 M CD1530 for these experiments. The gene expression level of *OSR1*, analyzed by qRT-PCR, was higher following treatment with AM580 or TTNPB than with ATRA, adapalene, or CD1530 (Figure 4H). Therefore, AM580 and TTNPB were most effective among the RAR agonists examined for inducing IM cells by the small molecule method.

### Activation of the BMP-Smad1/5 Signaling Pathways during IM Cell Differentiation by the Small Molecule Method

**Figure 5:**
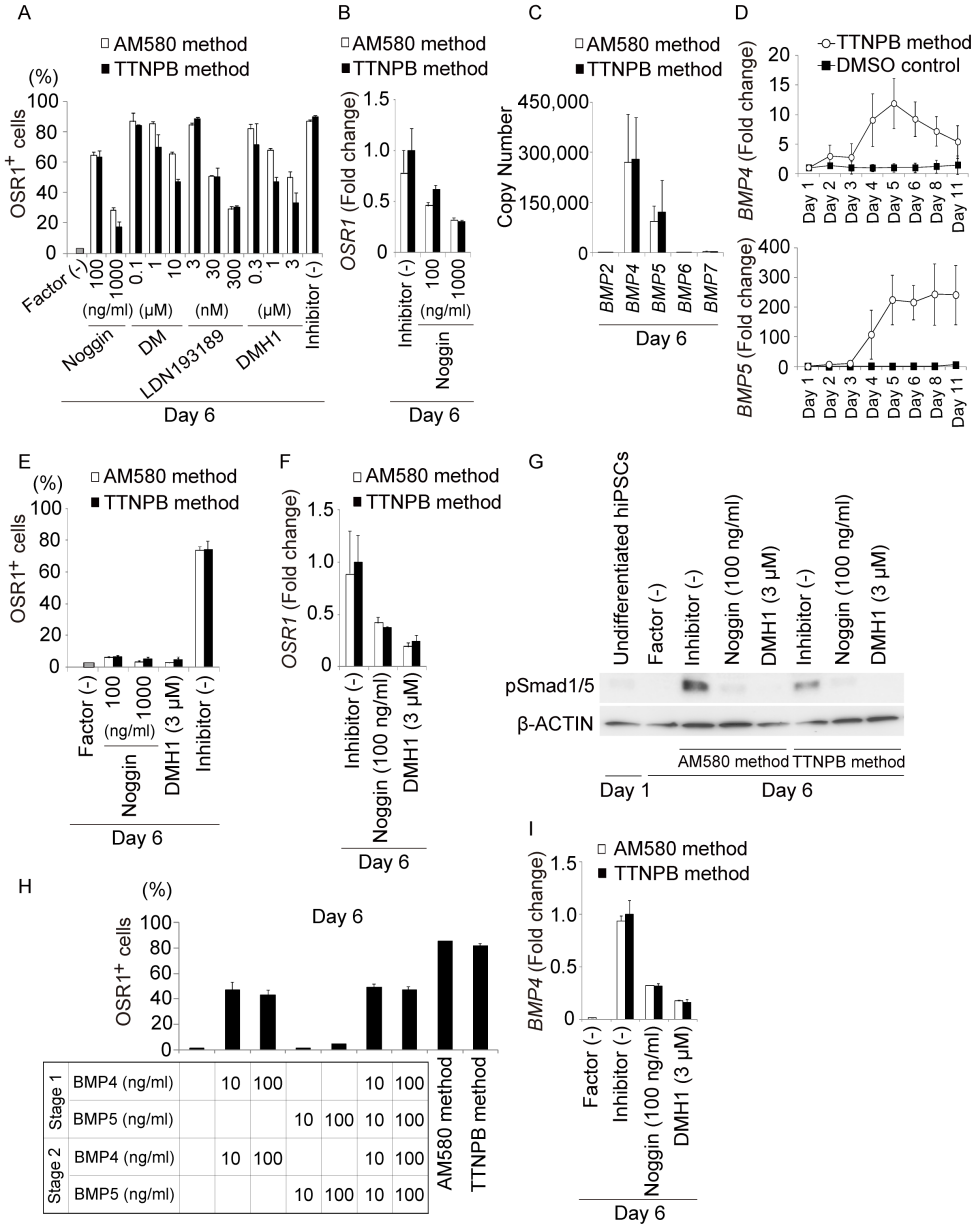
The BMP-Smad Signaling Pathway Regulates Development of IM. (A) Results of the flow cytometric analyses examining the effects of adding noggin, dorsomorphin (DM), LDN193189 and DMH1 during Stage 2 on the induction of OSR1^+^ cells on culture day 6 in the AM580 and TTNPB methods. (B) Results of qRT-PCR analyses showing mRNA expression of *OSR1* on culture day 6 of the AM580 and TTNPB methods, with or without noggin. (C) Copy numbers of *BMP-2*, *-4*, *-5*, *-6*, and *-7* expressed in differentiation cultures. (D) Time course of *BMP-4* and *BMP-5* mRNA expression in the TTNPB method. OSR1-GFP knock-in hiPSCs prior to treatments were used to normalize the data. (E and F) Effects of adding noggin or DMH1 during Stages 1 and 2 on the induction of OSR1^+^ cells and the *OSR1* expression levels analyzed on culture day 6. (G) Results of Western blot analyses examining phosphorylation levels of Smad1/5 in differentiation cultures of the small molecule method, with or without noggin or DMH1 added to Stages 1 and 2. (H) OSR1^+^ cell induction on culture day 6 in the small molecule method and following treatment with various combinations of recombinant BMP-4 and BMP-5 proteins. (I) Results of qRT-PCR analyses showing *BMP-4* mRNA expression in differentiation cultures on day 6 of the small molecule method, with or without the addition of noggin or DMH1. Factor (-) indicates the rate of induction of OSR1^+^ cell (A and E), the phosphorylation level of Smad1/5 (G) and the mRNA expression level of *BMP4* (I), on day 6 of the differentiation culture without growth factors or small molecules. OSR1-GFP knock-in hiPSCs obtained on day 6 following treatment of the TTNPB method without inhibitors were used to normalize the data shown in (B), (F) and (I). The data in (A–F, H, I) are means±SD of three independent experiments (n = 3). The data in (G) are representative of three independent experiments.

To further examine the differentiation mechanisms involved in the generation of IM cells by the small molecule method, we examined the effects of 18 signaling pathway inhibitors added individually during Stage 2 of the TTNPB method (Figure S4). Three days of culture with inhibitors of the BMP and Smad1/5/8 signaling pathways, including noggin (a BMP antagonist), dorsomorphin (an ALK2/3/6 inhibitor), LDN193189 (an ALK2/3 inhibitor), and DMH1 (an ALK2 inhibitor), reduced induction of IM cells in a dose-dependent manner (Figure 5A and Figure S4). qRT-PCR analyses revealed that addition of noggin inhibited up-regulation of *OSR1* gene expression (Figure 5B), indicating that BMP signaling pathways might be involved in differentiation of IM cells. To determine which BMP ligands are expressed during the induction process, we generated standard curves for the PCR products encoding five BMP ligands (BMP-2, 4, 5, 6, and 7), and analyzed the copy number of each ligand in cDNA samples of hiPSC-derived OSR1^+^ cells on day 6. *BMP-4* was the most abundantly expressed, and *BMP-5* showed the second highest expression (Figure 5C). Their expression levels gradually increased in a time-dependent manner until culture day 5. Interestingly, expression of *BMP-4* began to increase during Stage 1, around culture day 2 (Figure 5D), suggesting that the small molecule method with CHIR99021 and TTNPB activates *BMP-4* expression and Smad1/5/8 signaling pathways prior to day 3.

When noggin or DMH1 were added beginning on day 1 of the small molecule method, both OSR1^+^ cell differentiation and *OSR1* gene expression decreased more than when these factors were added at Stage 2 (Figures 5A, B, E and F). Immunoblot analyses detected high levels of Smad1/5 phosphorylation in cell lysates of hiPSC differentiation culture on day 6 of the small molecule method with CHIR99021 and AM580 or TTNPB. Phosphorylation levels were substantially attenuated by adding noggin or DMH1 to the culture (Figure 5G).

We next assessed the efficacy of treatment with BMP-4 or BMP-5 alone for inducing OSR1^+^ cells from hiPSCs. Induction was increased by approximately 45% after five days of treatment with 10 or 100 ng/ml BMP-4. In contrast, treatment with BMP-5 did not lead to any significant induction of OSR1^+^ cells, and no synergistic effects of BMP-4 and BMP-5 were observed, although both BMP-4 and BMP-5 were abundantly expressed in the differentiation cultures (Figure 5H). These results indicate that BMP-4, but not BMP-5, plays a crucial role in IM differentiation from hiPSCs, and that the mechanisms underlying the small molecule method include activation of the Smad1/5 signaling pathways through increased BMP-4 expression.

To further elucidate the regulatory mechanisms involved in increasing BMP-4 expression, we used qRT-PCR analysis to examine the effects of adding noggin or DMH1 on the expression of *BMP-4*. We found that the expression levels of both *BMP-4* and *OSR1* were reduced by adding BMP inhibitors (Figures 5F and I), suggesting that expression of *BMP-4* is regulated not only by the treatment with CHIR99021 and AM580 or TTNPB, but also by BMP-4 that is secreted from the induced cells, in a positive feedback manner.

### The Small Molecule Method Directly Generates IM Cells without the Mesendoderm Step

**Figure 6:**
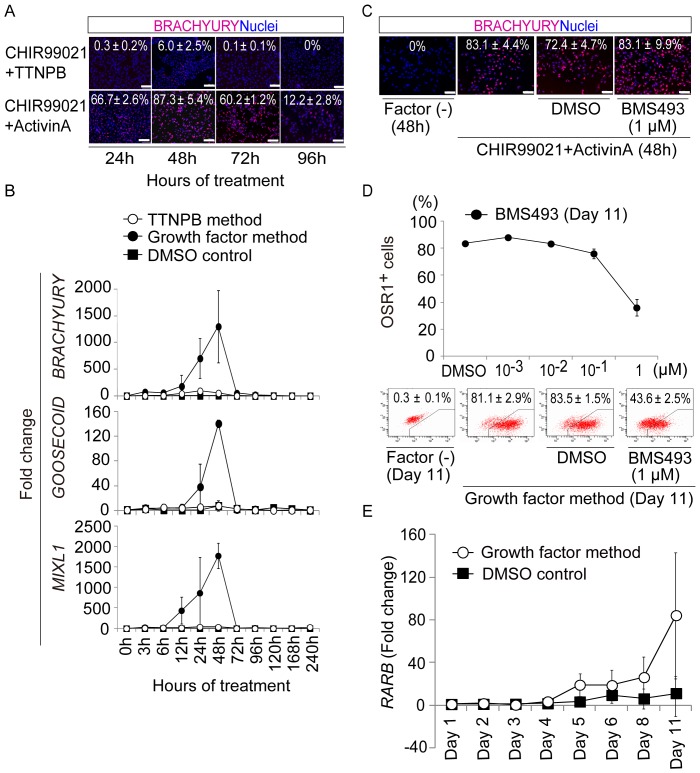
The Small Molecule Method Can Produce IM Cells without the Mesendoderm Step. (A) Induction of BRACHYURY^+^ mesendoderm cells generated from OSR1-GFP knock-in hiPSCs (3D45) after 24, 48, 72, and 96 hrs of treatment with CHIR99021 and TTNPB or activin A. (B) Results of the qRT-PCR analyses showing mRNA expression of the mesendoderm markers, *BRACHYURY*, *GOOSECOID*, and *MIXL1*, in differentiation cultures from the growth factor and TTNPB methods. (C) Effects of adding a RAR inhibitor, BMS493, to differentiation cultures of 3D45 cells treated with CHIR99021 and activin A, analyzed by anti-BRACHYURY immunostaining. (D) Induction of OSR1^+^ cells on culture day 11 of the growth factor method, with or without addition of BMS493 at various concentrations. (E) Results of qRT-PCR analyses showing expression of *RARB* mRNA. 3D45 cells on day 1, before treatment, were used to normalize the data shown in (B) and (E). The data in (A–E) are means±SD of three independent experiments (n = 3). Scale bars, 100 m.

In the small molecule method, peak induction of OSR1^+^ cells occurs around culture day 6, compared to day 10 in the growth factor method [19] (Figures 2A, B and Figure S2A). We tested the hypothesis that treatment with CHIR99021 and TTNPB generates mesendoderm cells faster than treatment with CHIR99021 and activin A by examining differentiation of BRACHYURY^+^ mesendoderm cells after 24, 48, 72, and 96 hrs of each treatment. Surprisingly, while treatment with CHIR99021 and activin A differentiated hiPSCs into BRACHYURY^+^ cells at an induction rate >80%, treatment with CHIR99021 and TTNPB resulted in a rate only around 6%, even at the peak time (48 hrs, Figure 6A). Then, we used qRT-PCR analyses to examine temporal expression patterns of three mesendoderm markers, *BRACHYURY*, *GOOSECOID*, and *MIXL1*, in differentiation cultures treated by the growth factor vs. TTNPB method. The expression levels induced by the TTNPB method were markedly lower than those induced by the growth factor method (Figure 6B), suggesting that the small molecule method produces IM cells without going through the mesendoderm step.

Notably, we found that the induction of OSR1^+^ IM cells by the growth factor method was significantly reduced by the addition of the RAR inhibitor, BMS493, without a concomitant reduction of BRACHYURY^+^ cell differentiation (Figures 6C and D). Furthermore, expression of *RARB* was detected on day 4 or later in the growth factor method (Figure 6E), which indicates that the induced RA signaling plays an essential role in IM differentiation, not only by the small molecule method but also by the growth factor method. As both CHIR99021 and BMP-7 were used during Stage 2 of the growth factor method, the combination of Wnt, RA, and BMP pathways are involved in the IM differentiation by both methods.

Overall, the results suggest that IM cells can be induced through activation of the Wnt, RA, and BMP pathways, without the mesendoderm step, and that the delay of the RA signal activation might result in the delayed generation of IM cells by the growth factor method as compared to the small molecule method.

## Discussion

Regenerative medicine is associated with high costs and enormous time requirements, because generation of large numbers of targeted cell types is necessary for clinical applications, such as cell therapy, and the efficiency of cell differentiation is generally low. Therefore, rapid, efficient, and relatively low-cost methods for differentiating hiPSCs/ESCs into target cell types are highly desirable. Previous studies differentiated hiPSCs/ESCs into renal lineage cells by combined treatment with activin A, BMP, and RA, but did not report induction rates [30], [31]. We recently developed a method for quantifying the induction rate of IM cells by generating a reporter hiPSC line for OSR1, and subsequently developed a differentiation method for IM cells using growth factors, with an induction rate >90% [19]. The goal of the present study was to identify small molecules that provide similarly high induction rates, but that are less expensive and more consistently effective than growth factors. We have identified two small molecules, AM580 and TTNPB, that are potent inducers of IM differentiation from hiPSCs, and developed a small molecule differentiation method, using just two chemicals, that is more rapid, more consistently effective, and less expensive than the growth factor method.

The IM cells generated using the small molecule method exhibited a developmental potential to further differentiate into cells of IM-derivative organs, such as the kidneys, adrenal cortex and genitalia both *in vitro* and *in vivo*, and form three-dimensional renal tubular structures in an organ culture setting. These findings were similarly observed in the IM cells generated using the growth factor method; however, the frequency of tubular formation in the organ culture samples was much higher in the samples obtained using the small molecule method (approximately 50%) than in those obtained using the growth factor method (approximately 5%) [19]. In addition, the small molecule method more robustly generated IM cells than the growth factor method in terms of the cell number yielded, which is advantageous for the development of regenerative medicine strategies (Figures 2B, C and Figures S2A, B, S5).

Mouse embryonic tissues at the gastrulation stage are capable of RA synthesis, which occurs preferentially in the node and primitive streak [50], suggesting that RA signaling pathways play some roles in early development. RA treatments have been used to induce pronephric tissues *in vitro* from an animal cap [23]–[25], and to induce renal lineage cells from mESCs [26]–[28] and hESCs/iPSCs [30], [31]. A recent report has described the involvement of signaling through RAR, but not through RXR, in the mESC differentiation into IM cells [29]. However, the roles and mechanisms of RA signaling in the differentiation of renal lineage cells have not been fully elucidated, especially in human.

Results of the present study demonstrated that signals through RAR also play an essential role in human IM cell differentiation, that RARβ, one of the three RAR subtypes is involved in the induction process, and that one of the downstream players is the BMP-Smad1/5 pathway, which is known to regulate cell fate decisions of mesoderm tissues [32], [33]. Furthermore, the BMP signaling inhibitors, noggin and DMH1, inhibited IM differentiation and *BMP-4* expression, suggesting that positive feedback mechanisms in the BMP signaling pathways are activated when IM is generated. We also showed that TTNPB and AM580 most efficiently induce IM cells among the retinoids we examined.

It has been reported that all mesoderm and endoderm tissues, including IM, are derived from a common mesendoderm precursor *in vivo* [51] and *in vitro* [52]. We first aimed to generate IM cells through the mesendoderm and confirmed that treatment with CHIR99021 alone can induce the differentiation of mesendoderm cells. However, we accidentally found that the addition of TTNPB to CHIR99021 in stage 1 blocked the differentiation of the cells into the mesendoderm. The resultant small molecule method directly induced the differentiation of IM cells without the mesendoderm step by activating the Wnt, RA and BMP signaling pathways. Furthermore, we confirmed that RA signaling plays an essential role in IM differentiation, but not mesendoderm differentiation, in the growth factor method, although the activation of the RA pathway was delayed compared to that observed in the small molecule method. Therefore, we reasoned that the generation of IM cells using the small molecule method requires less time than that performed using the growth factor method (Figure 2B and Figures S2A, S5). Our results also suggest that target cells, including IM cells, can be directly generated from hiPSCs when all of the factors required for differentiation are provided by chemical compounds.

## Conclusion

The small molecule method can be used to rapidly, efficiently and consistently produce IM cells from multiple hiPSC/ESC lines without the mesendoderm step at a relatively low cost. This differentiation method may serve as a powerful tool for elucidating the mechanisms of mesoderm and kidney development as well as supplying cell sources for the development of regenerative medicine strategies for treating CKD.

## Supporting Information

Figure S1 **Mesendoderm Cells Can be Induced by the Treatment with CHIR99021 Alone.** (A) Induction of BRACHYURY^+^ cells from OSR1-GFP knock-in hiPSCs (3D45) on culture days 2, 3, 4, and 5, with or without CHIR99021. (B) mRNA expression of the mesendoderm marker genes, *BRACHYURY, GOOSECOID*, and *MIXL1*, in 3D45 cells treated for three days, with or without CHIR99021. 3D45 cells on day 1, before treatment, were used to normalize the data. The data shown are means ± SD of three independent experiments (n = 3). Scale bars, 100 µm.
(TIF)

Figure S2 **The Small Molecule Methods Can Rapidly and Efficiently Produce IM Cells from hiPSCs.** (A) Induction of OSR1^+^ cells generated by the AM580, TTNPB, and growth factor methods. (B) Numbers of OSR1^+^ and total cells induced by the AM580 method. The data shown are means ± SD of three independent experiments (n = 3).
(TIF)

Figure S3 **The Small Molecule Methods Can be Applied to Multiple hiPSC/ESC Lines.** Expression levels of *OSR1* were analyzed by qRT-PCR and compared for five hiPSC lines (3D45 [201B7], 201B6, 253G1, 253G4, and 585A1), and three hESC lines (khES1, khES3, and H9), treated for five days by the AM580 or TTNPB method, and an hiPSC line (3D45), treated for 10 days by the growth factor method. The samples of undifferentiated hiPSCs or hESCs on day 1 before the treatment of each hiPSC or hESC line were used to normalize the data. The data shown are means ± SD of three independent experiments (n = 3).
(EPS)

Figure S4 **Effects of 18 Inhibitors on Differentiation of OSR1^+^ IM Cells by the Small Molecule Method.** Effects of adding 18 signaling pathway inhibitors on induction of OSR1^+^ cells generated by the TTNPB method. The inhibitors were added to Stage 2. The data are means ± SD on culture day 6 of three independent experiments (n = 3).
(EPS)

Figure S5 **Schematic of the Differentiation Methods for inducing IM Cells from hiPSCs/ESCs.** The two differentiation protocols used to induce IM cells from hiPSCs/ESCs are shown: small molecule and growth factor methods. Note that the small molecule methods induce IM cells more rapidly than the growth factor method.
(EPS)

Table S1 **Binding Constants and Transactivation Properties of the Retinoids Used in the Present Study.** Kd values of the six retinoids are shown for the RARα, RARβ, RARγ, and RXRα receptor isotypes.
(PDF)

Table S2 **Primer Sequences Used in This Study.**
(PDF)

Table S3 **Antibodies and Lectins Used in This Study.**
(PDF)

Table S4 **Growth Factors and Chemical Compounds Used in This Study.**
(PDF)
