# Supplementary figures and images for: Efficient and Rapid Induction of Human iPSCs/ESCs into Nephrogenic Intermediate Mesoderm Using Small Molecule-Based Differentiation Methods
Source: PLoS One. 2014 Jan 15;9(1):e84881. doi: 10.1371/journal.pone.0084881 (PMC3893162; doi:10.1371/journal.pone.0084881)

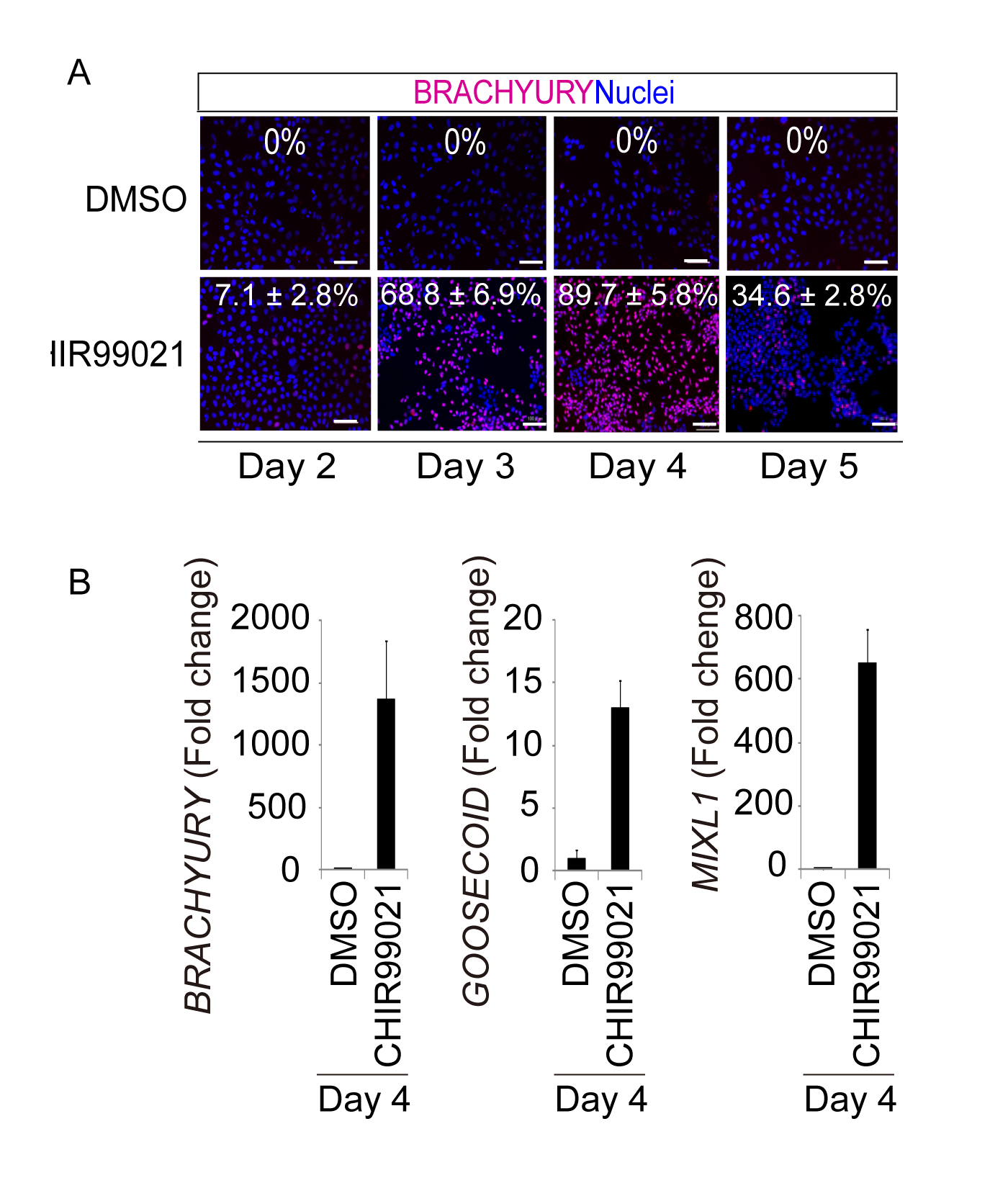

Supplement: Figure S1 — Mesendoderm Cells Can be Induced by the Treatment with CHIR99021 Alone. (A) Induction of BRACHYURY+ cells from OSR1-GFP knock-in hiPSCs (3D45) on culture days 2, 3, 4, and 5, with or without CHIR99021. (B) mRNA expression of the mesendoderm marker genes, BRACHYURY, GOOSECOID, and MIXL1, in 3D45 cells treated for three days, with or without CHIR99021. 3D45 cells on day 1, before treatment, were used to normalize the data. The data shown are means ± SD of three independent experiments (n = 3). Scale bars, 100 µm. (TIF) [file pone.0084881.s001.tif]

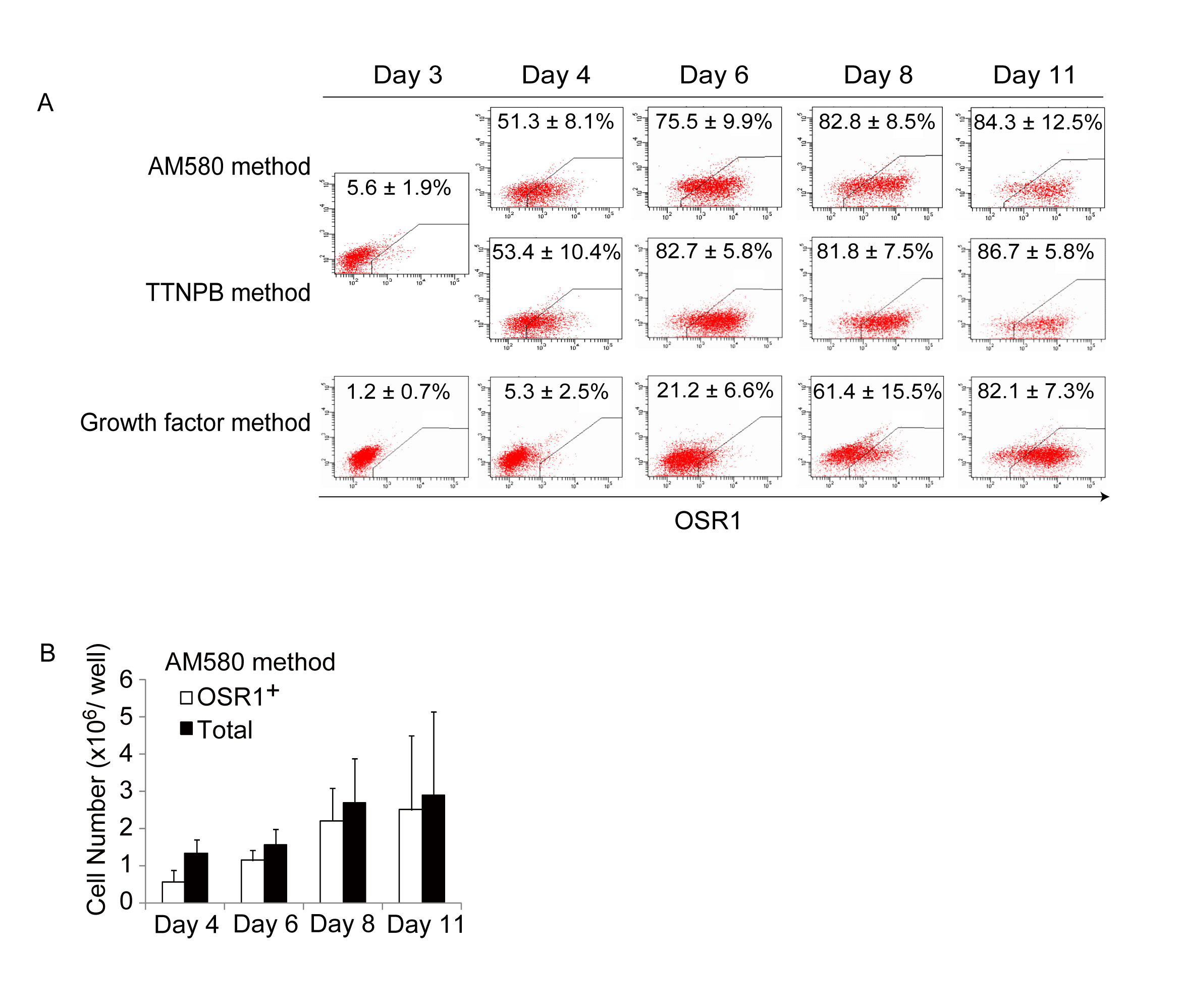

Supplement: Figure S2 — The Small Molecule Methods Can Rapidly and Efficiently Produce IM Cells from hiPSCs. (A) Induction of OSR1+ cells generated by the AM580, TTNPB, and growth factor methods. (B) Numbers of OSR1+ and total cells induced by the AM580 method. The data shown are means ± SD of three independent experiments (n = 3). (TIF) [file pone.0084881.s002.tif]
