# Supplementary material for: Efficient and Rapid Induction of Human iPSCs/ESCs into Nephrogenic Intermediate Mesoderm Using Small Molecule-Based Differentiation Methods
Source: PLoS One. 2014 Jan 15;9(1):e84881. doi: 10.1371/journal.pone.0084881 (PMC3893162; doi:10.1371/journal.pone.0084881)
Supplement: Table S1 — Binding Constants and Transactivation Properties of the Retinoids Used in the Present Study. Kd values of the six retinoids are shown for the RARα, RARβ, RARγ, and RXRα receptor isotypes. (PDF) [file pone.0084881.s006.pdf]

Retinoids	Action	Kd (nM)			
		RAR α	RAR β	RAR γ	RXR α
ATRA	Pan-RAR agonist	15.5～16	4.5～7	3	730
AM580 (CD336)	RAR α agonist	8～10	74～131	450	(No binding)
TTNPB	Pan-RAR agonist	3	3	3	
SR11237 (BMP649)	Pan-RXR agonist				100
Adapalene (CD271)	RAR β γ agonist	1100	34	130	(No binding)
CD1530	RAR γ agonist	2750	1500	150	
