# Supplementary material for: Efficient and Rapid Induction of Human iPSCs/ESCs into Nephrogenic Intermediate Mesoderm Using Small Molecule-Based Differentiation Methods
Source: PLoS One. 2014 Jan 15;9(1):e84881. doi: 10.1371/journal.pone.0084881 (PMC3893162; doi:10.1371/journal.pone.0084881)
Supplement: Table S2 — Primer Sequences Used in This Study. (PDF) [file pone.0084881.s007.pdf]

Gene name	Primer sequence	Size (bp)
hβ-ACTIN	CAATGTGGCCGAGGACTTTG<br>CATTCTCCTTAGAGAGAAGTGG	126
hRARB	CTGTGAGGGATGTAAGGGC<br>GTCTCCTTCTTTTTCTTGTTCCTGTC	195
hBMP2	TGTATCGCAGGCACTCAGGTC<br>CGGGTTGTTTTCCCACTCGTTTC	145
hBMP4	GACCACCTCAACTCAACCAACCA<br>GGCACCCACATCCCTCTACTA	184
hBMP5	CTCACCAGCGAAGGCATTACA<br>CAAAGACAAGCCAACCCACATC	240
hBMP6	AGCGTGGTGACAAGGGATG<br>GGGACTGGGTAGAGCGATTA	187
hBMP7	CAAGATAGCCATTTCCTCACCG<br>TCCGATTCCCTGCCCAAGT	257
**Mesendoderm**		
hBRACHYURY	AATTGGTCCAGCCTTGGAAT<br>CGTTGCTCACAGACCACA	112
hGOOSECOID	GAGGAGAAAGTGGAGGTCTGGTT<br>CTCTGATGAGGACCGCTTCTG	72
hMIXL1	TTGGTTCAAAGCTGGACTCA<br>CTGTCAGTCATGGCTCCTCA	107
**Ectoderm**		
hSOX1	CACAACTCGGAGATCAGCAA<br>GGTACTTGTAATCCGGGTGC	133
**Endoderm**		
hSOX17	CAGCAGAATCCAGACCTGCA<br>GTCAGCGCCTTCCACGACT	68

**Intermediate mesoderm**		
hOSR1	GCTGTCCACAAGACGCTACA<br>CCAGAGTCAGGCTTCTGGTC	137
hPAX2	AGATTCCCAGAGTGGTGTGG<br>GGGTATGTCTGTGTGCCTGA	264
hLIM1	TCATGCAGGTGAAGCAGTTC<br>TCCAGGGAAGGCAAACTCTA	148
hWT1	GGCAGCACAGTGTGTGAACT<br>CCAGGCACACCTGGTAGTTT	136
hEYA1	GGACAGGCACCATACAGCTACC<br>ATGTGCTGGATACGGTGAGCTG	189
hCITED2	CACCAATGGGCTGCACCATCAC<br>GCCGCTCGTGGCATTCATGTTG	157
hSALL1	AGCGAAGCCTCAACATTTCCAATCC<br>AATTCAAAGAACTCGGCACAGCACC	147
**Lateral plate mesoderm**		
hKDR	TGATCGGAAATGACACTGGA<br>CACGACTCCATGTTGGTCAC	131
**Metanephric mesenchyme**		
hSIX2	AGGAAAGGGAGAACAACGAGAA<br>GGGCTGGATGATGAGTGGT	132
hHOXD11	TGGAACGCGAGTTTTTCTTT<br>CTGCAGACGGTCTCTGTTCA	149
**Nephric duct and ureteric bud**		
hSALL4	CAGATCCACGAGCGGACTCA<br>CCCCGTGTGTCATGTAGTGA	109
hHOXB7	GTGGACTGTGGGTCTGGACT<br>GAACACGCGAGTGGTAGGTT	114

**Metanephric stroma**		
hFOXD1	TGCGGGTCCCTCTATTTATG<br>TAACGCCTGGACCTGAGAAT	190
**Gonad and adrenal cortex**		
hDAX1/NR0B1	CAAGGAGTACGCCTACCTCA<br>GCGTCATCCTGGTGTGTTC	131
hSF1/NR5A1	CAGGAGTTTGTCTGCCTCAA<br>GCACAGGGTGTAGTCAAGCA	126
hHSD3β2	GAGCCATTCCTGAAAAGAGC<br>GATGAAGACTGGCACACTGG	168
hGATA4	CTGTCATCTCACTACGGGCA<br>GGGAGACGCATAGCCTTGT	132
hGATA6	CATGACTCCAACTTCCACCT<br>ACTTGAGCTCGCTGTTCTCG	146
hLHX9	GCGAACCTCTTTCAAGCATC<br>TCCTTCTGAATTTGGCTCGT	164

Gene names and primer sequences (5'–3') for RT-PCR and qRT-PCR are shown.
