# Supplementary material for: Efficient and Rapid Induction of Human iPSCs/ESCs into Nephrogenic Intermediate Mesoderm Using Small Molecule-Based Differentiation Methods
Source: PLoS One. 2014 Jan 15;9(1):e84881. doi: 10.1371/journal.pone.0084881 (PMC3893162; doi:10.1371/journal.pone.0084881)
Supplement: Table S3 — Antibodies and Lectins Used in This Study. (PDF) [file pone.0084881.s008.pdf]

Antibody & Lectin	Dilution rate	Manufacturer & Clone
BRACHYURY	1 : 200	R&D systems, MAB2085
PAX2	1 : 50	Covance Research Products, PRB-276P
WT1	1 : 50	Santa Cruz Biotechnology, C-19
SALL1	1 : 200	Perseus Proteomics Inc., PP-K9814-001
LIM1	1 : 50	MILLIPORE, AB3200
SALL4	1 : 100	Abnova, M03
HSD3β	1 : 50	Santa Cruz Biotechnology, 37-2
GATA4	1 : 50	Santa Cruz Biotechnology, C-20
GATA6	1 : 50	Santa Cruz Biotechnology, H-92
LTL	1 : 200	Vector laboratories, B-1325
AQP1	1 : 100	MILLIPORE, AB3272
AQP2	1 : 50	Santa Cruz Biotechnology, N-10
PODOCALYXIN	1 : 50	R&D Systems, MAB1658
DBA	1 : 100	Vector laboratories, FL-1031
CYTOKERATIN8	1 : 200	Abcam, ab9023
αSMA	1 : 200	SIGMA, A2547
E-CADHERIN	1 : 200	BD Pharmingen, 610181
Laminin	1 : 200	SIGMA, L9393
Human nuclear antigen	1 : 100	STEMCELLS, STEM101
Human mitochondria	1 : 200	Abcam, ab3298
pSmad1/5	1 : 200	Cell Signaling, 9516

A summary of antibodies and dilutions used for immunostaining analysis
