# Supplementary material for: Efficient and Rapid Induction of Human iPSCs/ESCs into Nephrogenic Intermediate Mesoderm Using Small Molecule-Based Differentiation Methods
Source: PLoS One. 2014 Jan 15;9(1):e84881. doi: 10.1371/journal.pone.0084881 (PMC3893162; doi:10.1371/journal.pone.0084881)
Supplement: Table S4 — Growth Factors and Chemical Compounds Used in This Study. (PDF) [file pone.0084881.s009.pdf]

Factors	Manufacturer
ATRA	Sigma Aldrich
AM580	Santa Cruz Biotechnology
TTNPB	Santa Cruz Biotechnology
Adapalene	Sigma Aldrich
CD1530	Tocris Bioscience
LE135	Santa Cruz Biotechnology
BMS493	Sigma Aldrich
MM11253	Santa Cruz Biotechnology
SR11237	Tocris Bioscience
UVI3003	Santa Cruz Biotechnology
Wnt3a	StemRD
CHIR99021	Axon Medchem
Activin A	R&D Systems
BMP4	R&D Systems
BMP5	R&D Systems
BMP7	R&D Systems
Forskolin	R&D Systems
SB435142	Cayman Chemical
ALK5 inhibitor (TGF-β RI kinase inhibitor II)	Wako
A-83-01	EMD Bioscience
Noggin	PeproTech
Dorsomorphin	Merck
DMH1	Tocris Bioscience
LDN193189	Axon Medchem
IWP-2	Santa Cruz Biotechnology
H-89	Sigma Aldrich
Bisindolylmaleimide I	Merck
DAPT	R&D Systems
KAAD-cyclopamine	R&D Systems
PD173074	Wako
JAK inhibitor I	Santa Cruz Biotechnology
CCG-1423	R&D Systems

LY294002	R&D Systems
PD98059	Cell Signaling Technology
